# Evolution of Electricity by the Human Body

**Published:** 1842-02-19

**Authors:** William H. Muller

**Affiliations:** Pittsburgh


### *Evolution of Electricity by the Human Body.*

BY WILLIAM H. MULLER, M. D.

To the Editors of the Medical Examiner.

GENTLEMEN,—The object of this communication is to announce to you a singular and perhaps important discovery, which your correspondent chanced to make a few days since. I say discovery, because I am not aware that exactly the same phenomena have appeared to any one before. I have ascertained that a comparatively large amount of electricity can be developed in *all* persons, I may say, of both sexes, and all ages, by muscular contraction in a certain position, and only in such a position, together with a certain degree of temperature. If these conditions are not observed, no effect is produced.

In the 5th vol. of Tilloch's Magazine, (old series) which is to be found in the Franklin Library of Philadelphia, there is an article on animal electricity with original experiments by a Mr. Hemmer of the Electoral Academy of Sciences at Manheim. From these experiments, which were made in 1786, on thirty persons of different sexes and ages, and amounted to upwards of two thousand four hundred in number, Mr. Hemmer came to the following conclusions:—That electricity is common to all men; that it is sometimes negative, and oftener positive, and sometimes wanting; that it is produced without friction of the clothes, and was evolved from the naked body; that its quality is altered by certain circumstances, and changed from one to the other kind by sudden and violent motion—from positive to negative by cold—or lessened in amount by it; that continued mental exertion increased the positive electricity, &c. &c. If I am not very much mistaken, it was stated in the account, that Hemmer employed an electrical condenser. If he was obliged to use this instrument, it proves that the quantity he was able to obtain at any time must have been exceedingly small. His great number of experiments also shows that this was most probably the case. They must also have required no little care in their performance, or have been somewhat difficult to repeat, or he would not have confined himself to thirty persons in performing two thousand four hundred and twenty-two experiments. The results, however, which I have obtained, are so striking, and so quickly and easily performed, that no doubt can be entertained, both that the source of the electricity is the human body, and that, with perhaps a few exceptions, it can be developed in every individual. I was led to the discovery in the following manner:

I had several times attempted with a common gold leaf electrometer to verify the results of Mr. Hemmer's experiments by standing insulated for a length of time, with and without clothing, and then touching the electrometer, but I never found any indications of electricity. I at length thought I would try whether any was evolved from a limb when in that state of numbness called "asleep." My electrometer was on the mantel-piece. I sat before the fire on a chair with my arm over the back, so that the nerves were pressed upon. When it had become numb, I rose hastily and applied my finger to the cover of the electrometer, The leaves flew instantly to the sides of the glass, and I thought my theory fully verified. I was mistaken as to the cause, however; for, on rising again, applying the other hand, which was in its natural state, the same result took place. I soon found that this phenomenon depended on my rising quickly from a sitting posture. I then tried this with other persons. Some succeeded at the first trial, others failed in the first or second attempt, but succeeded as soon as they placed themselves in the proper position. I have given this experiment a fair trial with twenty persons of different age and sex, in different rooms, and with complete success. A little girl of seven years has shown very strong electric powers. To cause a movement of the gold leaf of half an inch from the perpendicular, I call a weak manifestation of electricity. In a properly warmed apartment, I can, by partially rising from a chair, and sitting down again, alternately, cause a continual and violent vibration of the gold leaf to and from the side of the glass—often there is force enough to tear the leaf, causing it also to adhere to the sides of the glass. It is not always necessary for me even to touch the cover of the instrument; nearly as striking results will follow if I but bring my finger *near* the cover—say an inch or more.

I will state more particularly the circumstances attendant upon a successful trial of the experiment.

First. I have generally succeeded best before a good fire, or in any part of a room equally warmed throughout to about 75° or 80°. I have always failed when trying it at the *side* of the fire-place, or in the corner formed by the projecting chimney and fire-place, and the wall of the room; and always succeed by moving my chair more to the front of the fire, the electrometer in both cases standing on the corner of the mantel-piece. But there are singular exceptions to this. Thus, I have produced a rapid and strong divergence of the leaf in several rooms without fire, but then only when near the empty fire-place, the electrometer being on the mantel-piece, while on trying it some hours after in the same rooms, and apparently under the same conditions, no such effect followed. I have also, after constant success for two or three hours in a warm room, found the experiment suddenly to fail. Returning once from a walk of near an hour, I found it impossible to produce any motion in the gold leaf, though, before going out, I had produced a continual oscillation of it. I attribute these varying results to fatigue in some cases, and also to some change in the body or skin, or the air, preventing the evolution or discharge of electricity.

Secondly. I have satisfied myself that the developement of electricity is not owing to friction: because, on the one hand, I have succeeded two or three times with no article of clothing on but socks and boots, and once without even these; and very often—nearly as often as I have tried it—with only a loose cotton night-dress on; and, on the other hand, when dressed, although the body and limbs may be moved slowly or suddenly in every direction but the one above mentioned, so as to produce any amount of friction, the gold leaf will remain motionless. I have also, when undressed, and insulated, with one hand on the electrometer, rubbed the surface of the body with flannel and cotton without causing the slightest effect on the gold leaf.

Thirdly. As to to the position requisite. This is as follows: Place the electrometer on the mantel-piece over a good fire. Take a common sized chair, with a back to it, of such a height, that the feet resting on the floor, the thighs shall be horizontal. Sit towards the front edge of the chair, and lean back, so as to have the trunk of the body quite relaxed; then rise quickly and touch the cover of the electrometer. The leaf, or leaves, will scarcely fail to indicate the presence of electricity. If the first trial should fail, it will be owing to the non-observance of some of the above mentioned conditions; a second or third *must* succeed. The electrometer may also be placed on a table before the fire; the experimenter seated, as described, on a chair near it, may place his hand on the cover, and then, after leaning back, he should lean a little forwards and rise quickly, or but partially assume the erect position. At the instant of rising, and very often at that of sitting down after having risen thus, the electrometer will indicate a large amount of electricity. I have charged a jar with as much as could be detected by the instrument, by thus alternately rising and sitting. I have not, however, been able to cause the leaf to move more than a quarter of an inch by applying a jar thus charged by half a dozen risings and sittings; though, by keeping the finger on the electrometer while I thus rose and sat, I could, as I said before, cause a continual flight of the leaf to and fro through an inch and more. I have hitherto found my own electricity positive, and I have a suspicion that the electricity is different according as I rise up or sit down. This shall be decided by future experiments.

It is indispensable that the chair be neither too high nor too low. If the chair with which I succeed, when in its proper position, be turned on its side, making it lower, and I then sit down and rise, no effect follows. Neither have I succeeded by rising from a rocking chair. At the suggestion of my friend, Mr. Swartzwelder, the effect of sitting on pillows, as upon non-conductors, was tried, and I found that, insulation aside, the *yielding* nature of these articles diminish the effect. If the chair be placed on pillows, and also the feet, or the experimenter sit on a pillow placed on the seat or against the back of the chair, the effects on the electrometer are irregular, and, for the most part, null. Any position, in short, which does not call into action the proper muscles, or impedes their complete action, entirely prevents or lessens the developement of electricity. So, lowering the body so that the buttocks rest on the heels, and then rising and touching the instrument, will be as void of influence on the leaf as movement from any other position but the one described. Complete insulation by placing the legs of the chair in glass tumblers and the feet on pillows, seems to increase the electricity. After rising and sitting in quick succession 15 or 20 times, causing a constant to and fro movement of the leaf, it became less and less frequent and more weak until the effect ceased. Thus it would seem dependent on the strength of the system; for, after twenty such movements, no little fatigue is induced.

Such, then, are the results of my experiments, which I believe are entirely novel; for although it is generally known that electricity is, nay, *must* be evolved both in animals and vegetables by the vital processes, especially by the formation of carbonic acid gas, and may be detected under ordinary circumstances with delicate instruments; and though Prevost and Dumas think that they have shown that electricity is produced during muscular contraction, and Edwards has proved that the same bodies which do or do not conduct electricity, do or do not conduct the nervous power, yet no one has hitherto observed the relation that exists between bodily motion in a certain direction, and the copious evolutions of electricity. Upon the ultimate cause of this phenomenon it is not in my power as yet to throw any light—perhaps others may be more fortunate. When discovered, it may serve to explain some of the mysteries of mesmerism. At present I can only come to the conclusion that this manifestation of electricity depends either upon the contraction of certain muscles that act together only when a person rises up from a certain sitting posture, or returns to the same after rising; or upon the pressure of some internal organ by these muscles; or upon some peculiar action in the brain, spinal marrow, or plexuses of the sympathetic during such muscular effort, combined in all cases with a certain temperature or other condition of the skin. It is certainly intimately connected with the state of the nervous system. Some have thought the spleen to be an electric organ; it may or may not be. I shall pursue my investigations, and if any thing novel turns up, it shall be the subject of another communication. Meanwhile I should be glad if some one would repeat these experiments and make known the result.

*Pittsburgh*, February 9th, 1842.

